# Remarks on Dysentery

**Published:** 1851-09

**Authors:** Wm. Kenney

**Affiliations:** Millersburgh, Kentucky


ART. V.—*Remarks on Dysentery.* By WM. KENNEY, M. D., of Millersburgh, Kentucky.

Without any wish to claim precedence for a favorite practice in any disease over that of other members of the profession, I would respectfully submit the following remarks on Dysentery.

For some twenty years past, in the generality of diseases of this country, prevailing at whatever season, sooner or later, a characteristic irritability of the gastro-enteric system presents itself, demanding a modification of the previous plan of treatment. In the inflammations of the splanchnic cavities, in the form of fever, diarrhea, or dysentery, this is the prominent condition in the disease. It offers a barrier to the modes of practice in use with the older writers, especially to venesection, the sheet anchor of the profession in a former age. Being thus debarred the use of this potent remedy, the next in order is mercury with opiates, constituting a favorite combination, to be resorted to on the decline of disease.

Dysentery, according to my observation is, in its incipient stage, more or less marked by nausea and the vomiting of a muco-bilious matter, or a colored watery fluid, with a pale clay-colored diarrhea of a cream-like consistence, attended with much uneasiness and pain about the umbilicus. The nausea and vomiting measurably subsiding, the operations become more frequent, thin, and cider-like in character, with an increased amount of pain before and after each operation.

As the disease progresses, without much arterial excitement or nervous disturbance, it gradually or hurriedly runs into the form of a well developed dysentery. This stage attained, the tormina and tenesmus become extremely distressing, the disposition to stool is frequent and urgent, the evacuations are muco-sanguineous, muco-purulent, or sero-sanguineous, and more or less offensive to the smell.

A febrile movement now presents itself, varying from that of a mild form to one of much gravity and seriousness. The pulse rises from 100 to 120 in the minute, a tender and tympanitic state of the abdomen comes on, superadded to which is a considerable amount of nervous disturbance, with pain in the head and back, aching of the extremities; the patient becoming restless and exhibiting a disposition to delirium during the height of the exacerbation, with paucity of urine.

The disease now fully matured, with all the phenomena incident to this class of affections, passes on to resolution, or ends in death. The chronic form rarely supervenes upon an acute attack, nor is the fatality so common as to entitle it to rank among the more malignant diseases of this section of country.

But to the treatment. The plan, then, which in my experience most speedily allays the pain and irritation, arrests the progress of the morbid action in the mucous surface of the bowels, and determines to the skin, is, if consulted early, during the disposition to nausea and vomiting, with evidence of a vitiated state of the contents of the stomach, to administer an infusion of chamomile flowers to promote emesis and rid the stomach of its offensive contents.

This effected, and the stomach quieted, I am in the habit of prescribing the following:

℞. S. morph., gr. ss;
Tannin, gr. j;
Ipecac., ää;
Nit. potas., grs. v.

This, in the early stage of the less violent forms of the disease, with a strict observance of the horizontal posture, generally effects a cure. But if not called until a distressing tormina and tenesmus have come on, with frequent disposition to stool, the discharges muco-sanguineous, or muco-purulent, with a tender and tympanitic state of the abdomen, fever, and much nervous disturbance, results so desirable are not to be expected; but the patient is still to be kept in the horizontal position, and to resist every effort at fecal evacuations.

This period in the disease reached, without a hope of its immediate termination, and with increased vascular action and a disturbed state of the nervous system more predominant, we would continue the prescription altered as follows:

℞. Sulph. morph., grs. iv;
Tannin, grs. vj;
Ipecac, grs. jx;
Nit. potas., ʒj; to be divided into twelve parts, of which one is to be given every three or four hours, or after each operation, increasing or diminishing the proportion of morphia as the severity of the pain and symptoms may indicate.

The surface should be kept constantly bathed during fever; the face and head in cold water, to which might be added ice; the body and extremities in warm water, while we apply bags of scalded bran, or sacs of boiled corn to the abdomen.

For drinks, the patient may be allowed the moderate use of ice-water, and occasionally pellets of ice might be broken up and swallowed; but as a general thing, elm-water is the most preferable.

The situation of the patient somewhat improved, the operations diminished in frequency, and the tormina less distressing, the interval between the administration of the anti-dysenteric powder might be gradually prolonged; but should the tormina and tenesmus, with disposition to stool, continue, great relief would follow the administration of an anodyne enema, after every other operation, to which might be added, if the sanguineous character of the excretions required it, from five to ten or fifteen grains of acetate of lead in solution.

The dysenteric discharges once arrested, the necessity for laxatives rarely occurs under five or ten days, provided the anti-febrifuge and refreshing applications of the cold and warm baths are kept up. Should it however recur, an enema, blue lick water, or an efferves-cing draught of Seidlitz water, at intervals of a few hours, would abundantly suffice for that purpose.

When that irritable state of the bowels, so frequently aggravated by cathartics, is controlled, and the secre-tions remain suspended, blue mass in combination with pulv. Dov. at night, will produce a fine effect, and in-sure rest.

Convalescence fairly commenced, the patient's diet, hitherto of a fluid and unirritating kind, may be improved by the addition of soups and the more digestible of the tender meats, with some gentle stomachic to promote digestion and his recovery.

This course, faithfully pursued, has given us every reason to be gratified with its adoption, in uncomplica-ted cases of the disease, and the good results following its practice warrant us in recommending it to practition-ers in whose limits dysentery may prevail.

July, 1851.

